# Ornamental Marine Species Culture in the Coral Triangle: Seahorse Demonstration Project in the Spermonde Islands, Sulawesi, Indonesia

**DOI:** 10.1007/s00267-014-0343-6

**Published:** 2014-08-01

**Authors:** Susan L. Williams, Noel Janetski, Jessica Abbott, Sven Blankenhorn, Brian Cheng, R. Eliot Crafton, Sarah O. Hameed, Saipul Rapi, Dale Trockel

**Affiliations:** 1 Bodega Marine Laboratory and Department of Evolution and Ecology, University of California at Davis, PO Box 247, Bodega Bay, CA 94923-0247 USA; 2 Mars Symbioscience Indonesia, Jl Kima 10 Kav. A6 Daya, Makassar, Sulawesi Selatan Indonesia; 3 Bodega Marine Laboratory and Population Biology Graduate Group, University of California at Davis, Davis, CA 95616 USA; 4 Present Address: Darden Aquasciences, C19-2, 1st Floor, Block C, Kepayan Perdana Commercial Centre, 88300 Kota Kinabalu, Sabah Malaysia; 5 Bodega Marine Laboratory and Graduate Group in Ecology, University of California at Davis, Davis, CA 95616 USA; 6 Bodega Marine Laboratory and Graduate Group in Applied Mathematics, University of California at Davis, Davis, CA 95616 USA

**Keywords:** Aquaculture, Coral Triangle, Indonesia, Ornamental species, Livelihoods

## Abstract

Ornamental marine species (‘OMS’) provide valuable income for developing nations in the Indo-Pacific Coral Triangle, from which most of the specimens are exported. OMS culture can help diversify livelihoods in the region, in support of management and conservation efforts to reduce destructive fishing and collection practices that threaten coral reef and seagrass ecosystems. Adoption of OMS culture depends on demonstrating its success as a livelihood, yet few studies of OMS culture exist in the region. We present a case study of a land-based culture project for an endangered seahorse (*Hippocampus barbouri*) in the Spermonde Islands, Sulawesi, Indonesia. The business model demonstrated that culturing can increase family income by seven times. A Strengths Weaknesses Opportunities Threats (SWOT) analysis indicated good collaboration among diverse stakeholders and opportunities for culturing non-endangered species and for offshoot projects, but complicated permitting was an issue as were threats of market flooding and production declines. The OMS international market is strong, Indonesian exporters expressed great interest in cultured product, and Indonesia is the largest exporting country for *H. barbouri*. Yet, a comparison of Indonesia ornamental marine fish exports to fish abundance in a single local market indicated that OMS culture cannot replace fishing livelihoods. Nevertheless, seahorse and other OMS culture can play a role in management and conservation by supplementing and diversifying the fishing and collecting livelihoods in the developing nations that provide the majority of the global OMS.

**Electronic supplementary material:**

The online version of this article (doi:10.1007/s00267-014-0343-6) contains supplementary material, which is available to authorized users.

## Introduction

There is increasing interest in culturing ornamental marine species (hereafter ‘OMS’) in light of the dramatic increase in the trade over the past two decades (Tlusty 2002; Cato and Brown 2003; Wabnitz et al. 2003; Moorhead and Zeng 2010; Olivotto et al. 2011; Rhyne et al. 2014). Due to the high value of OMS compared to food fish, OMS culture can provide much-needed livelihood support in developing nations (Norris and Chao 2002; Tlusty 2002; Pomeroy and Balboa 2004; Bazilchuk 2008). The vast majority of OMS are exported from Indonesia and the Philippines in the Coral Triangle (Balboa 2003; Wabnitz et al. 2003; Rhyne et al. 2014). The Coral Triangle, which stretches from Australia, north to the Philippines, and west to Malaysia, supports the highest coral reef and seagrass biodiversity on earth, but it is subject to multiple environmental threats (Bruno and Selig 2007; Burke et al. 2012). The OMS trade itself is a threat to Coral Triangle ecosystems when over-collection and destructive collection using cyanide occur (Kolm and Berglund 2003; DeVantier et al. 2004; Lunn and Moreau 2004; Shuman et al. 2005; Tissot et al. 2010), or non-native species are released in the region (Moore and Ndobe 2007). In recognition of these factors, the Coral Triangle Initiative (CTI) for Coral Reefs, Fisheries and Food Security set more effective management and sustainability of trade in reef ornamental species and live reef fish as a target for 2020 (Fidelman et al. 2012).

OMS culture could potentially reduce threats to coral reef ecosystems by decreasing trade reliance on vulnerable wild-caught ornamental species, which are challenging to manage through conventional fisheries strategies (Tlusty 2002; Olivotto et al. 2011; Fujita et al. 2013; Rhyne et al. 2014). Diversification of economic opportunities is essential to reducing the severe fishing pressure in the Coral Triangle and other developing regions (Cochrane 2000; Allison and Ellis 2001; Pollnac et al. 2001; Pomeroy et al. 2006; Newton et al. 2007; Salayo et al. 2008; Peterson and Stead 2011). Furthermore, culture is imperative for species such as corals and seahorses listed by the Convention on the International Trade in Endangered Species (CITES) (Evanston et al. 2011; Cohen et al. 2013; Rhyne et al. 2014). If OMS culture leads to diversification of livelihoods in the Coral Triangle, it could also serve as one element in Integrated Coastal Management strategies for net gains in conservation and human welfare (Clifton 2003, 2009; Webb et al. 2004; Pomeroy et al. 2005; Sievanen et al. 2005; White et al. 2005; Hill et al. 2012; Salafsky et al. 2011; Rhyne et al. 2014).

Technological advances in culture systems and controlling the life histories of desirable OMS have made their culture increasingly feasible (Moorhead and Zeng 2010; Job 2011; Olivotto et al. 2011). Despite these advances and the potential value for coastal management and conservation efforts, there have been relatively few studies of actual OMS culture in the Coral Triangle. These studies examined the culture or potential to culture clownfishes, seahorses, and invertebrates including corals (Pomeroy and Balboa 2004; Reksodihardjo-Lilley and Lilley 2007; Koldewey and Martin-Smith 2010; Ferse et al. 2012b; Rhyne et al. 2012a). Knowledge about culturing, familiarity with it, and successful demonstration all significantly influence whether OMS culture will be adopted, livelihoods will diversify, and conservation and management gains will accrue (Salayo et al. 2008; Ferse et al. 2012b). To this end, successful demonstration projects are needed.

**Fig. 1:**
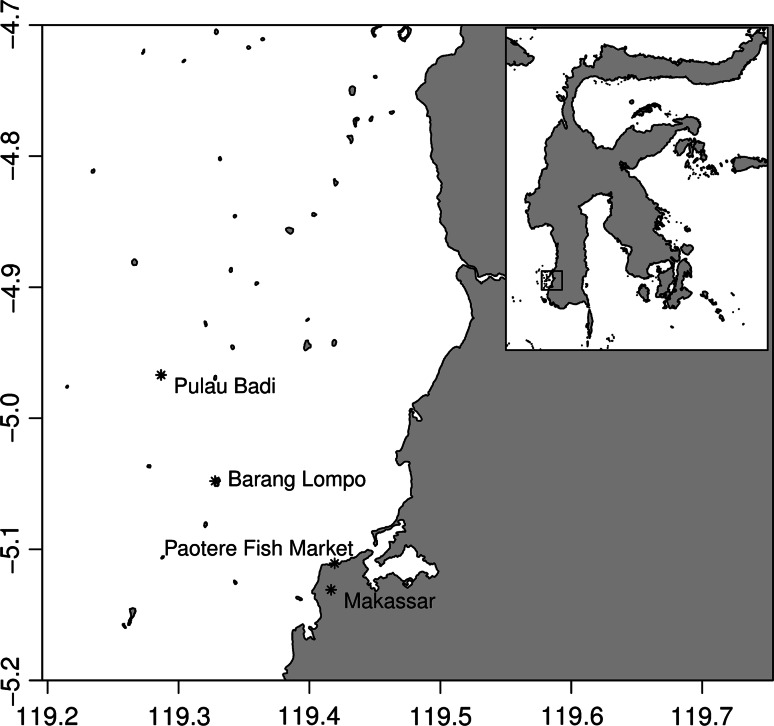
Map of southwest Sulawesi showing Pulau Badi (location of the kuda laut project) in the Spermonde Islands, Makassar, and the Paotere fish market

To address this gap in practical knowledge, we provide a case history of a demonstration project for OMS culture in the Spermonde Islands (hereafter ‘Spermondes’) off southwest Sulawesi, Indonesia (Fig. 1). The intrinsic isolation of island communities such as the Spermondes can strongly shape their response to management plans, particularly no-take zoning, and their willingness to depart from fishing activities (Webb et al. 2004). The Spermondes are representative of many locales within the Coral Triangle where fishing historically and presently provides the major livelihood, with few alternatives (Ferse et al. 2012a, b). The Spermondes are densely populated, water-limited, and agriculture is not a viable livelihood. Communities in the Spermondes depend on dwindling fisheries for food and income to send their children to school (Pet-Soede et al. 1999, 2001, 2011; Ferse et al. 2012b); islanders report that life and fishing have not been good since the 1960s. Fishermen face higher risk as they must travel farther and dive deeper for valuable species (Reksodihardjo-Lilley and Lilley 2007; Máñez and Ferse 2010). Fishermen are also indebted to their fishing patrons who are the middlemen in the supply chain to the local and international markets (Ferse et al. 2012a). The reefs and seagrass beds are under high threat from illegal bomb or blast fishing and pollution (Edinger et al. 1998; Pet-Soede and Erdmann 1998; Nadiarti et al. 2012). The marine habitats are too degraded to attract significant international ecotourism, with few exceptions. Although no-take areas were selected in each Spermondes village as part of Indonesia’s highly developed and supported Coral Reef Rehabilitation and Management (COREMAP) program, implementation of no-take areas and community-based management plans has not been fully successful (White et al. 2005; Clifton 2009; Glaser et al. 2010; Radjawali 2012). Non-fishing economic activities, such as aquaculture, have been recommended as a means to improve the success of coastal management as well as human welfare (Radjawali 2012).

Taken together, the factors cited above provide a strong rationale to attempt OMS culture in the Spermondes. The OMS pilot project we describe is based on culturing seahorses (‘kuda laut’ in Bahasa Indonesia, specifically *Hippocampus barbouri*). All seahorse species have been CITES listed in response to serious population declines attributed to the trades in ornamental species and traditional medicine, loss of seagrass habitat, bycatch, and small-scale fisheries (Martin-Smith and Vincent 2005; Hughes et al. 2009; Wiswedel 2012). Seahorses are ‘flagship species’ because they engender public support for conservation efforts (Vincent et al. 2011; Yasué et al. 2013). In general, CITES listing has the potential to facilitate a more environmentally sustainable OMS trade because it imposes export quotas, strict reporting, and fines, if the pressure to create and export product or over-collect broodstock is managed successfully (Tlusty 2002; Rhyne et al. 2014). Cultured seahorses are providing a greater portion of the seahorse trade since implementation of CITES export quotas in 2004 (Koldewey and Martin-Smith 2010; Evanston et al. 2011). To date, seahorse culture has been centered in developed nations but is shifting more to the developing nations where wild collection pressure is highest, such as in the Coral Triangle (Job et al. 2002; Koldewey and Martin-Smith 2010).

Our objective was to summarize the key lessons learned and the challenge and promise remaining in making OMS culture viable on small islands in the Coral Triangle. Our primary contribution is the case history of the seahorse culture demonstration project, but we place it in the broader context, from the seahorse and other OMS trade originating in Indonesia and entering California, which is the major entry point for Coral Triangle OMS in the United States, the major OMS-importing country (Balboa 2003; Wabnitz et al. 2003; Tissot et al. 2010), to key OMS exporters in the supply chain, and finally, the local dependence on food fish. These aspects integrate to form the framework in which to place the demonstration culture project.

## Methods

### OMS Culture on Small Islands in the Coral Triangle: Case History of a Kuda Laut Demonstration Project

A demonstration project to culture *H. barbouri* as a sustainable livelihood in a land-based system was established on the small Spermonde Island of Pulau Badi (Fig. 1). Several of the authors developed the business model for the project and have been involved from inception to the present, allowing a rare opportunity for a case history.

The profit-and-loss business model for a production unit was calculated for 2014 based on the three units in production, the allowable quota, and price paid by exporter *ex* producer. Establishment costs were calculated assuming a 10-year lifespan of the main assets of tanks, solar panels, and batteries. Energy was calculated separately because it can change greatly if the island is completely electrified. Operational energy costs include purchase of power from the grid (when operating) and running a generator when solar power is low.

Based on the demonstration project, we generalized the process of establishing OMS culture as a livelihood option in the Spermondes and the Coral Triangle into three consecutive stages: (1) culture development and early adoption, (2) scale-up to franchise, and (3) large-scale adoption including risk management of the supply chain integrity and product quality. We then summarized the important aspects of successful culture in a Strengths, Weaknesses, Opportunities, and Threats analysis (‘SWOT’, Valentin 2001). Strengths were identified as advantages in competition, knowledge, human, and financial assets. Weaknesses were assessed as disadvantages including vulnerabilities in human and financial resources, infrastructure, and markets. Opportunities focused on the global market and potential offshoot livelihoods. Threats included competition, quality control, and environmental factors.

### Indonesia OMS Exporters Interested in Cultured Kuda Laut

The kuda laut culturers sell directly to exporters. Exporters are critical links in the supply chain of OMS, and the OMS culture enterprise depends on them for transferring and air-freighting animals and export documentation, yet there is limited information on the OMS trade or culture from their perspective (Reksodihardjo-Lilley and Lilley 2007). We met with owners and/or executives of three of the largest, most experienced marine ornamental species exporter businesses in Indonesia (two on Bali, one in Jakarta) in March 2013 for a free-form discussion with each independently and a tour of their facilities. We specifically selected these exporters as opposed to a random sample because each was considered a ‘model’ exporter for the kuda laut project. Based on their high professionalism and experience, each had been approached initially about their interest in obtaining the licenses to sell the kuda laut product. One then obtained the licenses to sell seahorses from the project; the others remained interested in doing so. All had been in business for >25 years and two generations, and belonged to the Indonesian Coral, Shell, and Ornamental Fish Association (AKKII, Asosiasi Koral, Kerang dan Ikan Hias Indonesia). One exporter previously also had an import business in southern California, a major destination for OMS exports (see “Results” section). These exporters are ‘enlightened’ sensu Reksodihardjo-Lilley and Lilley (2007) in that they provide training for their suppliers and have holding facilities that buffer offsets in supply and demand, allowing suppliers a more continuous income. The discussions were conducted in English and Bahasa Indonesia, and lasted until exporters decided that they had nothing more to impart (minimum 2 h each).

### Indonesia’s OMS and Live Seahorse Trade

Seahorse and OMS culture must be considered both in the local Spermondes context as well as the international OMS trade originating in Indonesia. We thus gathered data on OMS exported from Indonesia to the United States, which is the largest importer of Indo-Pacific OMS, the majority of which enter through California (Balboa 2003; Wabnitz et al. 2003; Tissot et al. 2010). We obtained data on OMS imported from Indonesia into the ports of San Francisco and Los Angeles, California, USA, through a Freedom of Information request to the US Fish and Wildlife Service (USFWS)’s Law Enforcement Management Information System (LEMIS) for 2009, the most recent year for which data were considered complete by the agency. To supplement LEMIS records, we also observed a USFWS inspection at San Francisco International Airport (SFO) of a typical OMS shipment from Indonesia and obtained the invoice (exporter and importer information redacted). To our knowledge, the analysis is the most recent one for this major sector of the global OMS trade. For example, Rhyne et al. (2012b) reported quantities of ornamental marine fish importations into the entire United States in 2005 but not by country. Our data provide the first update of quantities of Indonesian OMS in the trade since Wabnitz et al. (2003), which reported fewer ornamental marine fishes imported globally from Indonesia between 1997 and 2002 than for California alone in 2009 (see “Results” section).

We also searched the CITES Trade Database (http://www.unep-wcmc-apps.org/citestrade; United Nations Environmental Programme World Conservation Monitoring Centre, Cambridge, United Kingdom. Accessed 1 April 2014) to identify all import and export records for the genus *Hippocampus* from 2004 to 2012, which covers the first year of implementation for the species (starting in May) and the most recent year for which data are available. Only records designated with the importer term ‘Live’ were included in the analysis. Data were further parsed by combining the importer source codes ‘C’ (bred in captivity as defined by CITES) and ‘F’ (born in captivity to wild-caught parents as F1 or subsequent generations that do not otherwise fulfill definitions of ‘bred in captivity’) compared to ‘W’ (taken from the wild).

### OMS Culture: The Spermondes Food Fish Contrast

Culturing OMS cannot be evaluated fully without understanding the importance of food fishes and small-scale marine fishing integral to life in southwest Sulawesi. The Spermondes form the largest coral reef fishery in Indonesia, and fish is a major food source (Pet-Soede and Erdmann 1998; Budan Pusat Statistik, http://www.bps.go.id, accessed 10 April 2014). The fishery supplies both people in Makassar, the major city in southwest Sulawesi and where the catch is largely sold, and Spermondes islanders, who buy their daily fish back from Makassar as a consequence of the patron (‘middleman’)-client structure of the fishing industry (Ferse et al. 2012a). Makassar bills itself as a tourist destination for seafood, primarily fish. We sampled fishes in the Paotere fish market near Makassar, which is the largest of the two other fish markets in the region, for the quantity, diversity, and value of food fishes and numbers of people employed as vendors, to contrast with OMS culture. After a preliminary assessment visit to the market in 2012, we sampled the fishes being sold by randomly selected marine fish vendors on one day each in March (17 vendors) and September (25 vendors) in 2013 and March (25 vendors) 2014. We counted the total number of marine vendors at the market, photographed the fishes of each selected vendor, counted the number of individuals or estimated number if sold in baskets or piles, and asked vendors the price and where the fish were caught. Allen et al. (2003), White et al. (2013), Froese and Pauly (2013), and references therein were used for identification. We excluded tilapia and milkfish because they probably came from aquaculture.

## Results

### OMS Culture on Small Islands in the Coral Triangle: Case History of a Kuda Laut Demonstration Project

The first production unit was started in 2009 and commercial international sales of CITES-certified seahorses commenced in September 2011, for a total of 2,800 animals sold through January 2014. This unit currently produces ~300 animals per month (=production capacity). A second unit is producing ~200 seahorses per month and is awaiting final inspection by LIPI (Lembaga Ilmu Pengetahuan Indonesia or the Indonesia Institute of Sciences, responsible for CITES oversight) for sales licensing. The third unit has limited production. Five more units are anticipated for establishment within a year because funding and demand exist (see below).

Profits and losses were calculated and then scaled to the production/unit of 200–400 animals per month (Table 1), based on the results of the three units currently in production. The income generated from a kuda laut production unit is substantial for the island families that own and operate each production unit and sell directly to exporters. At the allowable quota of 200 animals/month, price paid by exporter (Indonesian rupiahs Rp$30,000/animal), and exchange rate (Rp11,000 ~ US$1), the profit is roughly seven times the typical monthly income (~US$350/month profit versus < US$50; unpublished data) for a male head of a Pulau Badi household. The highest current production capacity is 300 animals/month; thus, approval to increase the quota would yield a more robust business model by allowing for temporary production shortfalls, e.g., major equipment failures.

**Table 1:** Profit (‘laba’) and loss (‘rugi’) statement and production costs in Indonesian rupiah (Rp) for *H. barbouri* cultured on Pulau Badi

Profit/loss	Per month	% Total cost			
Income					
Sales (total income)	6,000,000	100			
Expenditures					
Establishment cost/depreciation					
Facilities	166,667	8.0			
Energy system	364,583	17.5			
Equipment	233,333	11.2			
Licenses	108,333	5.2			
Initial broodstock	104,167	5.0			
Energy system					
Maintenance	58,333	2.8			
Operational	455,000	21.8			
Consumables					
Maintenance	83,333	4.0			
Breeding system	58,333	2.8			
Feed system	145,833	7.0			
Water quality, disease management	100,000	4.8			
Administration, licensing, certification	205,000	4.8			
Total expenditures	2,082,917	100			
Net profit	3,917,083				

Production costs	Total cost/month	Production costs per animal			
# animals produced		150	200	300	400
Energy	513,333	3,422	2,567	1,711	1,283
Consumables	592,500	3,950	2,963	1,975	1,481
Depreciation	977,083	6,514	4,885	3,257	2,443
Total costs		13,886	10,415	6,943	5,207
Price/animal		30,000	30,000	30,000	30,000
Profit/animal		16,114	19,585	23,057	24,793
Income/month		2,417,083	3,917,083	6,917,083	9,917,083

The Profit/Loss statement is for one production unit at the current production quota of 200 animals per month. ‘Price per animal’ (30,000Rp) was based on €2/animal currently paid by exporter. The Production costs demonstrate how the profit changes with the allowable quota. The exchange rate used is approximately Rp11,976 to US$1 (June 2014)

In the demonstration project, the start-up costs were provided by the private business (Mars Symbioscience). Facilities expenditures included concrete in-ground, fiber, and biofilter tanks (Table 1). Water system expenditures included filters, pipes, pumps and fittings, ultraviolet sterilizer, and installation. The energy system expenditures included solar panels, DC inverter, battery and charger, controller, electrical panel, cables, and installation. Consumables included holding tanks, aerations, feed *(Artemia*; mysid and plankton collection and storage equipment), feed system equipment, water filters, water testing, disease analyses, ice, and maintenance costs. Administrative costs included travel to obtain licenses and certification, office equipment, printing, and telephone. There were also unaccounted administrative costs that were, and must continue to be, subsidized by the private business because at present, islanders cannot navigate the current complicated permitting process.

Below, we detail the critical elements in each phase of culture development, which are generally applicable to OMS culture in the Coral Triangle. The major findings are then summarized in a SWOT analysis (Table 2; see also Supplementary Table S1).

**Table 2:** SWOT analysis of kuda laut culture in the Spermonde Islands, southwest Sulawesi

Strengths	Weaknesses
Land-based	Complicated licensing process
Low capital and running costs	Production decline
Community-based	Lack of scientific input
Culturer-owned	Lack of experience
Direct supply chain (culturer to exporter)	Limited visibility for project results
Private business involvement	
Community–Private–Government cooperation	

Opportunities	Threats
Growing international demand	Shifting preferences for species
Increasing Indonesian exporter interest	Quality control
Technology transfer to other species	Market flooding
Culturing more species	
Ecotourism	

#### Phase 1: Technological Development and Early Adoption

The first phase involved the establishment of the full cycle production system, engagement of local families, training, system optimization, and demonstration of feasibility. Collaboration among the islanders, private business (Mars Symbioscience), and academic researchers was essential, and private business provided the start-up capital. Repeated discussions between village leaders and representatives of the private business that provided the financing and technical support were important to convince islanders to risk the unknown. The process to assess interest and select culturers was managed by the village head but as production proceeded and relationships were built, senior people in different parts of the island joined the headman in managing discussions. Eight families were initially interested in culturing seahorses, but the protracted licensing period winnowed the interest to one initially. All three current culturers were fishermen who still fish but not on a daily basis. Each small (~8 m × 5 m) culture unit was constructed in the family’s yard area.

An important feature of the project was that the economic success was directly attributable to the early adopters because they owned the production units and supplied kuda laut directly to the exporters, which is a major departure from the patron (middleman)-client system characterizing fishing in the Spermondes (Ferse et al. 2012a). Ownership is an important factor determining adoption and success of supplemental or alternative livelihoods as features of coastal management plans (Ferse et al. 2012a). Another factor influencing willingness to culture was exposure to non-traditional life beyond the islands, which characterized all early adopters.

Another critical factor in this early phase was the dedication of owners and their ability to solve problems. Not surprisingly, differences existed among owners. The first adopter was dedicated, acted independently to solve problems, and ran trials to determine the best culturing methods. In contrast, the owner of another unit currently producing too little to be successful had delegated operation to a family member. This culturer was not capable of addressing the suspected root causes of the low production, which included plumbing not to specification, breeding inhibited by warm water, and disturbance of animals by mosque noise. If production remains low, the unit will be converted to culturing less sensitive OMS. The first two units have already begun limited culture of other OMS, and they also a grow-out species provided by government hatcheries (e.g., clownfishes, *Amphiprion* spp.; blue devils *Chrysiptera cyanea*; barramundi cod, *Cromileptes altivelis*). Adding species has leveraged the basic infrastructure with a few incremental assets to provide additional income at reduced overhead per Rp of income.

In the start-up phase, private business promoted the transfer of academic expertise in fish aquaculture to kuda laut culture and provided the investment capital, logistical experience necessary to complete projects in Indonesia, and technical expertise required to develop a reliable, renewable, low-cost supply system. The latter was important because electrical power is not typically available during daylight hours (or at all on some islands). While wind and wave energy systems were also evaluated, a solar energy system was adopted to lower maintenance needs. Finally, sustainable feed systems had to be established to ensure availability when local wild harvest feeds (mysid shrimp) are in low supply. Collaborations with government research laboratories were established by the private business to develop sustainable feed systems, which will become increasingly important as more families adopt OMS culture as a livelihood opportunity.

The private business was also essential for overcoming the most challenging aspect of the project—linking the culturer directly to the exporter and obtaining the necessary licenses and permits (see results below on exporters). The licensing and CITES permit processing took more than 2 years to complete. The process included the acquisition of licenses to collect broodstock, keep the seahorses in captivity, commercially produce the seahorses under a specified quota allocation, undertake domestic sales, undertake export sales, and then finally obtain the CITES permit required for every individual shipment of seahorses.

#### Phase 2: Build-Out to Franchise

This phase concerns defining the technologies and business systems to enable others to duplicate the system, i.e., the franchise. Eventually, the experienced culturers can receive payment for providing technical support, which has been provided free to the current culturers and included a franchise manual detailing how to establish and operate a successful production unit. The present technology holders (early adopters) must develop the capacity to support technology transfer to help others efficiently and consistently produce a quality product. Capital must be available for additional start-ups and subsequent expansions into small businesses. The collector, exporter, and wholesale businesses must be able to connect to a growing network of small producers and provide market signals to manage supply and demand in reasonable balance. Infrastructure investments (particularly electricity supply and sanitation) by the government or other capable entities are important to reduce the individual investment and ongoing operating costs.

#### Phase 3: Large-Scale Adoption of OMS Culture

This phase will depend on the success of the preceding phases. Investment must be made in further development of breeding technologies and feed systems to expand the range of captive-bred species and to reduce the complexity of captive breeding production systems. Streamlining the various licensing and audit processes will be essential to managing small OMS businesses within, rather than outside, the regulatory framework.

There is an additional future challenge of flooding an essentially niche market with technology transfers. The risk of rampant production, a market ruined by oversupply, and lack of adherence to a legal supply must be carefully managed to avoid cheating and a decline in the market. A control system incorporating barriers to entry is essential to help manage supply and demand to limit boom and bust cycles, and to maintain the integrity and credibility of the supply chain and the product. Such a control system could include licensing if legally mandated. Certifications offer another potential control, but add potentially prohibitive costs (Shuman et al. 2004; Cohen et al. 2013; Rhyne et al. 2014).

Another model for a control system would be an entity to serve as an information bank for the market and demand. Such an entity could collect and distribute data on the number of shipments, the number of broodstock, aspects of technology, and verification of the number of culterers trained to operate and that the organisms traded are captive bred and not creatively disguised wild specimens. Culturers will know the number of broodstock and the quantities sold to customers, and thus the demand. The ideal entity to implement the control system would be a cooperative of all the stakeholders—culturers, exporters, government, universities, and non-government organizations. Presently, Mitra Bahari South Sulawesi (Ricci and Crawford 2012), a forum involving university, government, not-for-profit organizations, and business partners, could serve as this entity.

##### SWOT analysis

The SWOT analysis highlighted the importance of a land- and community-based enterprise with major continued input from private business (Table 2). Being land- and community-based was also an inherent weakness. Land is limited in the Spermondes, and islanders had little technical and business knowledge, which contributed to the major challenge of licensing and continued subsidization. On the other hand, opportunities to expand into different species and other islands and importantly, reduce the time to licensing, will be enhanced due to the cooperation between the community, producers, exporters, private business, and the government’s aquaculture research laboratories. An opportunity for an ecotourism venue was also identified.

The SWOT analysis also underscored declines in production as typical for captive breeding and culture. A hiatus in production occurred during the rainy season in one year perhaps due to pollution and a fall off in production occurred at the fourth generation possibly due to unconfirmed inbreeding depression. Addressing production declines requires better genetic and other scientific information, and collaboration of all parties for quick action when a decline is evident. All parties also need to address the major future threats of market flooding and quality control.

### Indonesia OMS Exporters Interested in Cultured Kuda Laut and Other Cultured Species

The success of OMS culture in the Spermondes also depends on factors constraining exporters of the product (Table 3). Similarly, to the kuda laut culturers, the exporters faced a complicated regulatory permitting process, which differed by the export destination. Europe required more permits than the United States, including health certifications, but the United States was very strict about invoices and inspections and levied heavy fines for typographical errors. The lack of a standardized taxonomy, particularly for corals, caused discrepancies between invoices and inspections of shipment contents resulting in stiff fines. An additional challenge was the slow permitting process in Jakarta, which supposedly takes 24 h but extends to several days, adding significant cost. The exporters also had no easy way of acquiring information on species under consideration for regulation in other countries. One exporter was surprised to learn that the lionfish (*Pterois volitans*) had invaded the Atlantic and Caribbean seas (Calado and Chapman 2006), and was unaware of the general problem of invasive ornamental species.

**Table 3:** Information provided by three major OMS exporters in Indonesia (Bali, Jakarta)

Exporter	1	2	3
# spp./shipment	1,000	300 fishes	50 fishes; 40 invertebrates
# Individuals/shipment	2,000	300 large–1,500 small	300–400 *Chromis* alone
# Shipments/year	1,000	720	20 (down from 40)
Destinations	Europe, US, AU	LAX primary, then MIA, Asia (Japan), Canada, Middle East (new market)	LAX; transship to JFK, MIA, etc.
CITES permits	Corals, seahorses, giant clam	Corals	Corals
Profitable species	Corals, seahorses (not much profit in fishes)	Fishes, corals, ‘dermata’ (invertebrates), damselfishes	Any; mostly sells damselfishes, angelfishes, butterflyfishes, clownfish (*Amphiprion ocellaris*)
# Staff	20	240	20
# Middlemen/collectors	>20	40–42	20 (down from 40)
Challenges	Strict invoicing/inspection (US)	Strict invoicing/inspection (US)	Strict invoicing/inspection (US)
	Multiple permits (Europe)		Multiple permits (Europe)
	CITES paperwork (Indonesia)	CITES paperwork (Indonesia)	Quota reductions (US)
	Non-standardized taxonomic references	Educating middlemen/collectors	Increasing regulation (US)
	Habitat to raise coral broodstock	Loss of Bali collection sites due to beach tourism	Cost of coral broodstock set-up
	Information on species considered for listing		Increasing fees (airlines, USFWS)
	Anticipating species considered for listing		

Exporters did not have a marketing strategy and were at the mercy of weather, availability, airline cargo cost volatility, a shift in consumer preferences away from aquariums to electronic entertainment, regulations, and particularly hobbyist preferences that vary by country. Exporters reported that Japanese favored frogfishes, while Americans had a distinct preference for rarity. Exporters indicated that distinctiveness always created a better product. Product quality was not an issue for these exporters who carefully maintained their clean facilities. Exporters demanded high-quality specimens from their middlemen and collectors, either through training (for up to 6 months) or by terminating employment upon receiving a sub-standard specimen. Exporters were seeking new, more remote collection sites (West Papua, Maluccas), which will lengthen the path to the destination and put pressure on previously unexploited populations.

Exporters emphasized that more cultured specimens are supplied by Indonesia than the Philippines and the Maldives, which rely more heavily on wild collections. To facilitate OMS culture, the Indonesia government established a broodstock habitat in the waters around Serangan Island, Bali, where a number of exporters grow-out corals and giant clams (*Tridacna* spp.) for export. However, exporters worried about the lengthy time to develop these products. For example, culturing *Tridacna* required up to 2 months to acquire permits, up to five years to develop the broodstock, and then up to 3 years to reach marketable size (5–7 cm). The cost of setting up an operation at this site (US$200,000–300,000) was prohibitive for some exporters. The Bali culture habitat is crowded, water quality is perceived to be declining, and disease is becoming a problem. Exporters also felt squeezed because collection sites have been reduced as the beach tourism industry has expanded on Bali.

Prospecting for new species to culture was important to exporters and depends on being able to control the species’ life history. Exporters identified currently popular candidates for culture, including damselfish (*Chrysopterus hemicyanea*), clownfish (*Amphiprion ocellaris*), mandarin fish (*Synchiropus splendidus*), the blue tang ‘Dory’ (*Paracanthurus hepatus*), and a coral (*Euphyllia glabrescens*). Angelfishes (Pomacanthidae) have been very lucrative (Supplementary Table S2) and have spawning behaviors similar to groupers (Serranidae), which have been successfully cultured for food. Exporters explained the current fads for ‘nanotanks,’ or very small home aquarium systems will favor culturing small-sized species.

Although one exporter worried whether his children could continue the family business, another neither observed nor anticipated a decline in business (see below). Finally, exporters indicated that the industry needed a focused, full-time commitment to assure the high product quality. When all of the above perspectives of the exporters are taken together, they indicate a scope for growth to develop OMS culture in the Spermondes.

### Indonesia’s OMS and Live Seahorse Trade

**Fig. 2:**
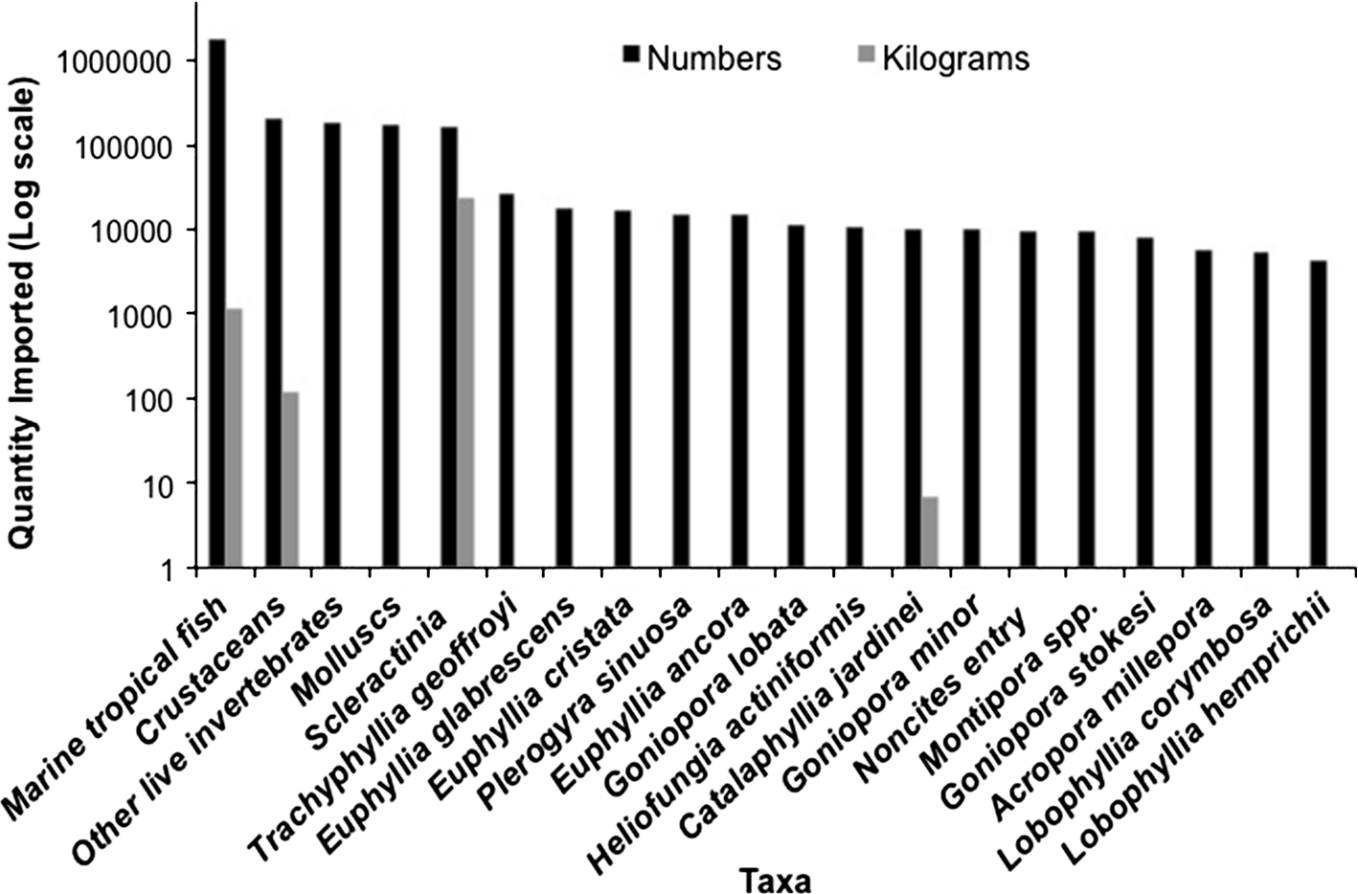
The major (by quantity) marine ornamental taxa imported into California (Los Angeles and San Francisco) in 2009 as recorded in USFWS’s LEMIS database. Quantities in each shipment can be reported by exporter as either weight (kg fresh mass) or # specimens. Each volume estimate is independent and likely an underestimate, as weight might be reported for only a portion of the number or vice versa. Many additional species imported in smaller quantities are not included

Indonesia has remained a major exporter of many OMS including seahorses, and California has remained a major destination of these exports. The results below indicate a high demand for Indonesian OMS and an increasing exportation of seahorses cultured in Indonesia, which indicates that market flooding is not an imminent threat. Thirty-seven Indonesian exporters shipped OMS through the Los Angeles and San Francisco International Airports to 36 importers in the USA in 2009, based on USFWS LEMIS records. In this year, over one million individual ‘tropical marine fishes’ were collected in and exported from Indonesia, followed by high numbers of ‘crustaceans,’ ‘molluscs,’ and hard corals (Fig. 2).

**Fig. 3:**
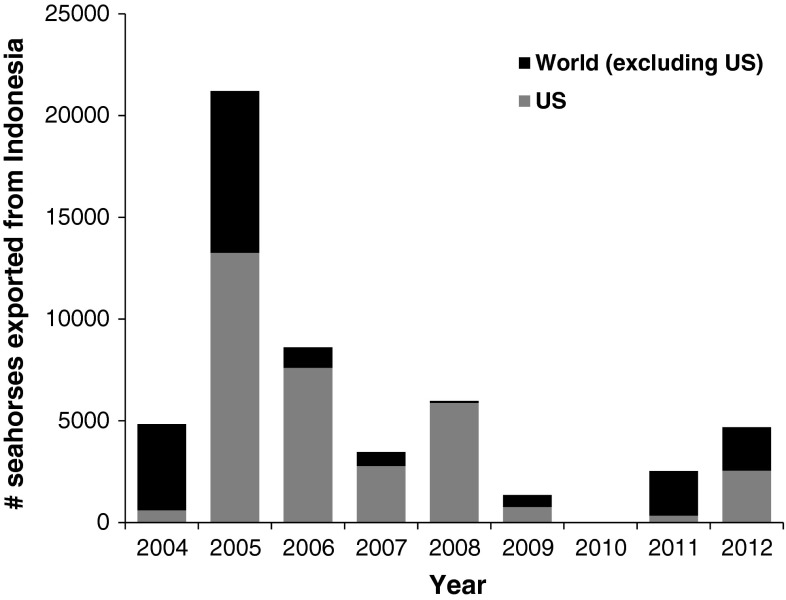
Numbers of seahorses (all taxa) exported from Indonesia to the United States and the rest of the world, as reported in CITES records. Data from 2004 begin in May

**Fig. 4:**
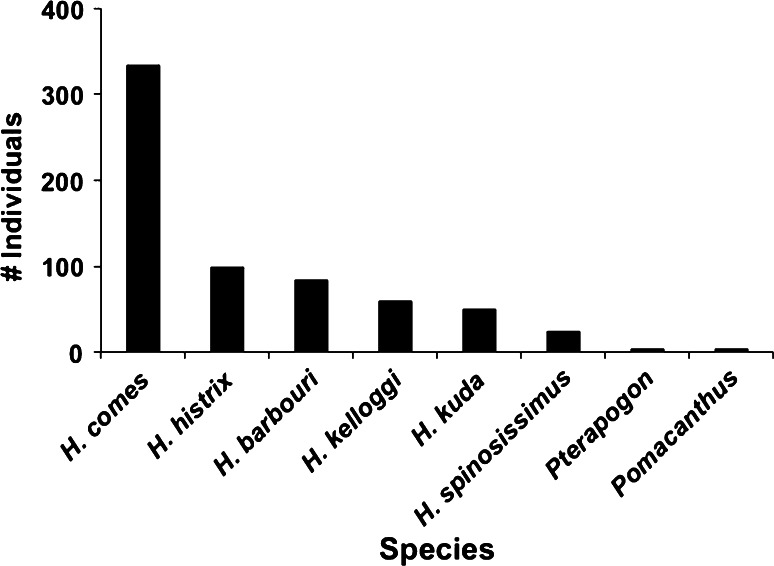
Quantities of seahorses (*Hippocampus*) and other marine ornamental fishes imported from Indonesia into California (Los Angeles and San Francisco) in 2009 and listed by species or genus (i.e., not as ‘marine tropical fish’) in USFWS’s LEMIS database. *H. comes* (‘tiger tail’ seahorse), *H. histrix* (‘spiny’ or ‘thorny’ seahorse), *H. barbouri* (‘Barbour’s’ seahorse), *H. kelloggi* (‘great’ or ‘Kellogg’s’ seahorse), *H. kuda* (‘common’, ‘estuary’, or ‘yellow’ seahorse), *H. spinosissimus* (‘hedgehog’ seahorse), *Pterapogon* (‘Banggai’ cardinalfish), *Pomacanthus* (angelfish)

Specifically, considering seahorses from 2004 to 2012, Indonesia exported 52,712 individuals representing at least 14 (plus ‘*Hippocampus* spp.’) of the 26 species of *Hippocampus* reported in the CITES database and accounting for 9 % of the global importations. The United States was the major world importer (62 %) of seahorses, and the majority of Indonesia’s exports (65 %) were to the United States (Fig. 3). Indonesia exported 5,700 *H.* *barbouri* or 82 % of the global live trade in this species from 2004 to 2012. The United States was the major importer of *H. barbouri* (65 % of global share), and Indonesia supplied the majority of these importations (85 %). Indonesia also supplied the majority of the global trade in *H. histrix* (91 %) and *H. spinosissimus* (60 %), and was the sole exporter of *H. denise*. Furthermore, all or the majority of the Indonesian seahorse exports to the United States were landed in California, comparing CITES to LEMIS records. In 2009, 634 of 763 individuals or 83 % of all Indonesia seahorse exports landed in California (Fig. 4). In 2012, 100 % of Indonesia’s seahorse exports landed in Los Angeles.

Across all years of data, most (83 %) of the *Hippocampus* exported from Indonesia were from wild sources (source code W: 66,975) and 14 % from captive sources (source code C: 7,084 individuals; F: 462 individuals), with the remainder labeled by other source codes. All of the captive-sourced exports from Indonesia occurred in 2009, 2011, and 2012 and included *H.* *barbouri*, *H. comes*, *H. kuda*, and *Hippocampus* spp. A shift to captive-sourced exports was evident by 2011, when 2,415 were exported, compared to 120 from wild sources. In 2012, 4,687 were coded either C or F as a source code (4,225 and 462, respectively), and none were from wild sources. Of the 2012 total, 720 were *H. barbouri*. The current allowable quota for each of the three kuda laut production units (200 animals per month) represents 3 % of the *Hippocampus* exported per month averaged across all years of available data. This comparison indicates scope for growth in kuda laut culture.

### OMS Culture: The Spermondes Food Fish Contrast

The sheer number of food fishes in the Paotere market indicated the importance of fishing in the Spermondes, and that OMS culture will not replace fishing as a livelihood. There were approximately 220 marine fish vendors in March 2013, 110 in September 2014, and 113 vendors in March 2014, of which we censused 8, 22, and 23 %, respectively, for a total of 65 vendors. Extrapolating from the total fishes counted and the number of vendors, we estimated that over 31,470–41,000 marine fishes were marketed daily (not accounting for the turnover) representing at least 140 taxa (Supplementary Table S2). Food fish species also exported as OMS were sold for a few US dollars apiece or even less (or US$1 - $3.60 maximum/kg) (Supplementary Table S2). The fish families with the highest diversity and abundances were the Serranidae (groupers/coral trouts/coral cods), Lutjanidae (snappers), and Lethrinidae (emperors/breams), each of which included species sold in the ornamental trade (Supplementary Table S2). All fishes were associated with coral and seagrass habitats, with the possible exception of flying fish and one dolphinfish each sold by a single vendor. The fishes were caught primarily at ‘the islands,” referring to the Spermondes, or “nearby and far,” “a day and night away,” and “one to six hours by boat,” although three vendors reported that fishes were caught in Kalimantan (Indonesian Borneo). Vendors identified six islands specifically but otherwise could not, because they bought from middlemen (the ‘patron’).

## Discussion

OMS culture in the Coral Triangle offers the promise to help reduce the environmental impacts of wild fish harvest and provide a much-needed economic supplement to fishing, which can enhance the success of environment management efforts in the region and meet a key goal of the CTI. The kuda laut case history demonstrates that OMS culture in the Coral Triangle can be successful but requires persistence, collaborations, and for now must be subsidized for technology and licensing and permitting. The start-up phase lasted 3 years before the first sales (but will likely shorten with accumulating experience). Kuda laut product is in high demand by exporters to supply an increasing international market, and villagers remain interested in culturing despite some hiatuses in production. Raising the allowable quota and adding other species up to the full capacity of existing fixed infrastructure (e.g., energy supply, pumps, space) would decrease production costs per animal. Government capitalization of infrastructure to the islands would also decrease production costs.

Despite the above scope for OMS culture expansion, for now, OMS culture should be viewed as a supplemental livelihood to fishing and collecting from the wild, based on our study and others (Pomeroy and Balboa 2004; Ferse et al. 2012a, b). Clearly, traditional fishing will continue as the local demand and international live food fish trade expand (Erdmann and Pet-Soede 1997; Mous et al. 2000; Scales et al. 2007; Pet-Soede et al. 2011; Radjawali 2012; Supplemental Table S2). To put OMS culture and trade into this perspective, on an annual basis, the food fishes sold in the Paotere market alone are roughly fivefold higher than the flux of ornamental marine fishes exported from Indonesia to California (the major global importer) and exceed the most recent estimates of exportations into the United States and globally (Wabnitz et al. 2003; Rhyne et al. 2012b), even accounting for uncertainties in numbers (Fig. 4).

By helping diversify livelihoods, OMS culture is one of multiple strategies for a robust socio-ecological system (Pomeroy et al. 2006; Ferse et al. 2012b; Foale et al. 2013; Rhyne et al. 2014). The socioeconomic benefits of the OMS trade were expressed poignantly by the exporters and witnessed as the economic advancement of the early adopters of kuda laut culture. Increasing knowledge about socio-ecological systems in the Coral Triangle combined with more pilot projects have led to a more realistic expectation about alternative livelihoods, including OMS and other aquaculture, as a management and conservation strategy (Pollnac and Pomeroy 2005; Sievanen et al. 2005; Hill et al. 2012; Ferse et al. 2012a, b). Even though not all fishers will switch to an alternative livelihood if and when available, livelihood diversity is important (Pollnac et al. 2001; Pomeroy and Balboa 2004; Sievanen et al. 2005; Cinner et al. 2008; Daw et al. 2012). Supplemental livelihoods can offer some poverty relief, which is important given that the value of fish in the local market has apparently changed little for over a decade (Pet-Soede et al. 1999; Supplementary Table S2). Even subsidized enterprises can lead to net management and conservation gains through supporting increased awareness and education (Pomeroy and Balboa 2004; Job 2005; Salafsky et al. 2011).

The kuda laut project demonstrated two critical factors for OMS culture in the Coral Triangle: 1) ownership assures direct profit accrual to the culturer/owner; and 2) involvement by private business has been crucial, including to directly link the supplier to exporter. Culturers can achieve a degree of autonomy and potential release from the debt, which is a significant departure from traditional patron-client system of fishing prevalent in at least the Spermonde Islands (Reksodihardjo-Lilley and Lilley 2007; Ferse et al. 2012a). By means of the sustained investment by private business, the owners of kuda laut units have not been subject to the limitations of loans and micro-credit systems such as those provided by the Indonesian government’s COREMAP program, and islanders acquired specialized skills (Brock 2013). Specialized skills further increase their ability to diversify their livelihoods as required for successful integrated coastal management (Pollnac and Pomeroy 2005). To date, management and conservation planning in the region has largely been in the hands of government and non-governmental organizations; however, the value that private business can bring to the process has been recognized (Salayo et al. 2008; Radjawali 2012).

The OMS trade, while not without environmental impacts, is less damaging than the wholesale destruction of coral reefs incurred by blast fishing. The net added value to conservation and livelihoods that culture can deliver versus wild harvest will vary by species and will change over time as the market for captive bred fish attracts larger premiums, the wild harvest catch comes under greater control, and the culture technology improves. The economic feasibility of culturing low value but high volume species will improve as the variety of products increase, and overhead costs can be shared with high value ones such as seahorses. Growing out species bred in large-scale hatcheries run by the government (e.g., barramundi cod) could potentially increase profits to islanders and is currently being attempted by the kuda laut culturers. The exporters expressed their keen interest in cultured fishes, as they, just as the fishermen, have been forced to collect farther afield as stocks are depleted, access to collection sites is reduced, and coastal water quality declines. Thus, a robust land-based culture of diverse ornamental species also has the potential to offset the ‘roving bandits’ syndrome, in which marine resources are serially depleted farther and farther away from local governances in response to global markets (Berkes et al. 2006). However, without good stock assessments, the effect on fishing pressure of OMS culture or any other management strategy will remain untested (Fujita et al. 2013).

The remaining issues for successful sustainable OMS culture have been addressed at length in the literature (Andrews 1990; Padilla and Williams 2004; Shuman et al. 2004; Bolton and Graham 2006; Olivotto et al. 2011; Dykman 2012; Cohen et al. 2013; Rhyne et al. 2014). They are often interrelated, and all require international action beyond the scope of kuda laut culture: the high cost of captive bred or cultured versus wild harvest products; the ability to control the life history; the traceability of product; and the risk of releasing invasive species. The kuda laut project demonstrated that OMS culture in the Coral Triangle brings an improved livelihood for some fishermen in the Spermonde Islands despite challenges remaining to be addressed. Ultimately, however, OMS culture is just one piece of a necessarily multi-faceted approach to integrated coastal management that also must include habitat protection and rehabilitation, diversification of both livelihoods and stakeholders, and strong fisheries management policies (Ferrol-Schulte et al. 2013).

## Electronic supplementary material

Below is the link to the electronic supplementary material.

Supplementary material 1 (DOCX 66 kb)

Supplementary material 2 (DOCX 159 kb)
