# Supplementary material for: Ornamental Marine Species Culture in the Coral Triangle: Seahorse Demonstration Project in the Spermonde Islands, Sulawesi, Indonesia
Source: Environ Manage. 2014 Aug 1;54(6):1342–55. doi: 10.1007/s00267-014-0343-6 (PMC4232751; doi:10.1007/s00267-014-0343-6)
Supplement: Supplementary file 1 — Supplementary material 1 (DOCX 66 kb) [file 267_2014_343_MOESM1_ESM.docx]

Supplementary Table 1. (word document, 65KB)

**Ornamental Marine Species Culture in the Coral Triangle:**

**Seahorse Demonstration Project in the Spermonde Islands, Sulawesi, Indonesia**

Summary Supplementary Table S1. Key phases and elements required for ornamental species culture to be successful as a sustainable alternative to fishing.

| Phases | Necessary Elements | Partners |
| --- | --- | --- |
| 1. Development phase | Willingness to risk unknown result  Capacity to culture (especially high-value species)  Initial investment capital  Market access  Licensing  Power supply | Former fishermen (early adopters)  Private business  Technology developers-   - Universities - research agencies - government |
| 2. Build-out to franchise | Clear demonstration of early adopter success  Display units producing and linked to market  Franchise manual  Expanded capital | Exporters  Customers  Financing institutions |
| 3. Large-scale adoption | All of above  Risk management of integrity and credibility  Management of value in expanding market  Control system and barriers to entry  Information database for monitoring market demand | All of above  Cooperative of stakeholders to verify quality (captive bred) and manage market information |
|  |  |  |
| 4. Prevention of aquarium releases into wild | Public education for consumers and hobbyists   - Invasive species risks - Appropriate disposal of unwanted species | Hobbyists  University  Importers/retailers  Trade associations |
