# Supplementary material for: Ornamental Marine Species Culture in the Coral Triangle: Seahorse Demonstration Project in the Spermonde Islands, Sulawesi, Indonesia
Source: Environ Manage. 2014 Aug 1;54(6):1342–55. doi: 10.1007/s00267-014-0343-6 (PMC4232751; doi:10.1007/s00267-014-0343-6)
Supplement: Supplementary file 2 — Supplementary material 2 (DOCX 159 kb) [file 267_2014_343_MOESM2_ESM.docx]

Supplementary Table 2. (word document, 145 KB)

**Ornamental Marine Species Culture in the Coral Triangle:**

**Seahorse Demonstration Project in the Spermonde Islands, Sulawesi, Indonesia**

Summary: Supplementary Table S2 provides families and species of market fishes and OMS fishes in a typical ornamental species shipment from Indonesia to San Francisco International Airport. Market values are compared to invoiced and online values.

Table S2. Part one: Marine market fishes. Families and species of marine fishes on sale by a total of 67 randomly-selected vendors at the Paotere fish market in March (M) 2013 and 2014 and September (S) 2013. Species indicated by * indicates home aquarium species on sale but not in random sample. The random samples included a minimum of 140 taxa, excluding small bait fishes, a conservative estimate because of turnover, fishes probably missed when displayed in mixed-species piles or baskets, and identification hindered by trauma or decay in many of the fishes. Market value: price quoted by vendors: invoice value: from Indonesia shipment into San Francisco airport in March 2012 (see second part of table). Online retail values: range (reflecting size) in online retail values for species in the major ornamental fish families sold in the market were obtained from the first and second sites from a Google search for “marine aquarium fish for sale” plus from PetCo’s online site (<http://www.petco.com/N_4294956696/Saltwater-Fish.aspx?CoreCat=LiveFishHPSaltwaterFish>). Searches were conducted between March and October 2013. Serranidae online prices did not include anthias and basslets (non-food fishes). Invoice value per individual reported for OMS shipment from Indonesia to San Francisco International Airport in March 2012.

Part two: Marine ornamental fishes. Families and species (n = 62), the numbers imported, the value ($US) per individual, and the size reported on the invoice of a typical shipment from Indonesia to San Francisco International Airport in March 2012. Names used by exporter indicated by ‘’.

| **Market Fishes** |  |  |  |  |  |  |  |
| --- | --- | --- | --- | --- | --- | --- | --- |
| Family and Species | Common Name | Mo | Market Value | Invoice Value | Online Retail Value | | |
|  |  |  |  |  | 1 | 2 | PetCo |
| Carcharhinidae | sharks |  |  |  |  |  |  |
| unidentified | sharks (finned) | M | $7.70 | na | na | na | na |
|  |  |  |  |  |  |  |  |
| Dasyatidae | rays |  |  |  |  |  |  |
| *Taeniura lymma* | bluespotted stingray | M | na | na | na | na | na |
|  |  |  |  |  |  |  |  |
| Clupeidae | sardines, sardinellas |  |  |  |  |  |  |
| *Amblygaster sirm* | spotted sardinella | M | <<$1 | na | na | na | na |
|  |  |  |  |  |  |  |  |
| Mullidae | goatfishes |  | <$1 | na | na | $24 | $19-30 |
| *Parupeneus barberinus* | dot dash goatfish | M, S |  |  |  |  |  |
| *Parupeneus multifasciatus* | many bar goatfish | S |  |  |  |  |  |
| *Mulloidichthys flavolineatus* | yellowstripe goatfish | S |  |  |  |  |  |
|  |  |  |  |  |  |  |  |
| Scombridae | tunas, mackerels |  | $4-11 | na | na | na | na |
| *Auxis rochei* | bullet tuna | M |  |  |  |  |  |
| *Euthynnus affinis* | mackerel tuna | M |  |  |  |  |  |
| *Grammatorcynus bilineatus* | doubleline mackerel | M |  |  |  |  |  |
| *Katsuwonus pelamis* | skipjack tuna | M |  |  |  |  |  |
| *Rastrelliger faughni* | island mackerel | M |  |  |  |  |  |
| *Thunnus obesus* | bigeye tuna | M |  |  |  |  |  |
| *Thunnus tonggai* | longtail tuna | M |  |  |  |  |  |
|  |  |  |  |  |  |  |  |
| Psettodidae | halibuts |  |  |  |  |  |  |
| *Psettodes erumei* | Indian halibut | S | na | na | na | na | na |
|  |  |  |  |  |  |  |  |
| Coryphaenidae | dolphinfish |  |  |  |  |  |  |
| *Coryphaena hippurus* | dolphinfish | M | $2 | na | na | na | na |
|  |  |  |  |  |  |  |  |
| Sphyraenidae | barracudas |  |  |  |  |  |  |
| *Sphyraena qenie* | blackfin barracuda | M | <$1 | na | na | na | na |
| *Sphyraena* sp. | bigeye or blackfin barracuda | S |  |  |  |  |  |
| *Sphyraena* spp. (two) | unidentified barracudas | M |  |  |  |  |  |
|  |  |  |  |  |  |  |  |
| Carangidae | jacks, trevallies, scads |  | $<1-8 | na |  |  |  |
| *Carangoides bajad* | orange-spotted trevally | S |  |  |  |  |  |
| *Carangoides gymnostethus* | bludger trevally | M, S |  |  |  |  |  |
| *Carangoides orthogrammus* | yellow-spotted trevally, pilotfish | M | na | na | na | $24-50 | $48-84 |
| *Caranx ignobilis* | giant trevally | M |  |  |  |  |  |
| *Caranx lugubris* | black jack | M |  |  |  |  |  |
| *Decapterus macarellus* | mackerel scad | M |  |  |  |  |  |
| *Decaptuerus russelli* | Russell’s mackerel scad | M |  |  |  |  |  |
| *Gnathanodon speciosus* | golden trevally | M | $7-8 |  |  |  |  |
| *Parastromateus niger* | black pomfret | M | $2 |  |  |  |  |
| *Selar boops* | oxeye scad | M | <$1 |  |  |  |  |
| *Selar crumenophthalmus* | bigeye scad | M | <$1 |  |  |  |  |
| *Selaroides leptolepis* | yellowstripe scad | M | <$1 |  |  |  |  |
| *Ulua mentalis* | longrakered trevally | M | <$1 |  |  |  |  |
|  |  |  |  |  |  |  |  |
| Exocoetidae | flying fishes |  |  |  |  |  |  |
| unidentified | flying fish | M | <<$1 |  |  |  |  |
|  |  |  |  |  |  |  |  |
| Gerreidae | silver biddies, mojarras |  |  |  |  |  |  |
| *Gerres acinaces* | longtail silver biddy | S | na | na | na | na | na |
|  |  |  |  |  |  |  |  |
| Labridae | wrasses |  | $2 | $2 | $14-60 | $20-40 | $13-46 |
| *Cheilinus* sp. | floral wrasse? | S |  |  |  |  |  |
| *Choerodon anchorago* | anchor tuskfish | S |  |  |  |  |  |
| *Coris gaimard** | coris wrasse | S |  |  |  |  |  |
| *Thalassoma hardwicke** | sixbar wrasse | S |  |  |  |  |  |
|  |  |  |  |  |  |  |  |
| Scaridae | parrotfishes |  | <$1-2 | $2 | $11 | $30 | $27 |
| *Chlorurus bleekeri* | Bleecker's parrotfish | M, S |  |  |  |  |  |
| *Scarus ghobban* | bluebarred parrotfish | M, S |  |  |  |  |  |
| *Scarus rivulatus* | surf parrotfish | M, S |  |  |  |  |  |
| *Scarus schlegeli* | yellowbar parrotfish | S |  |  |  |  |  |
|  |  |  |  |  |  |  |  |
| Ephippidae | spadefishes, batfishes |  | $5-12 | $6.90 | $30-80 | $30-40 | $19-36 |
| *Platax batavianus* | Batavia batfish | M |  |  |  |  |  |
| *Platax boersii** | golden batfish | S |  |  |  |  |  |
| *Platax circularis* | circular batfish | M |  |  |  |  |  |
| *Platax teira* | longfin batfish | M |  |  |  |  |  |
|  |  |  |  |  |  |  |  |
| Nemipteridae | monocle breams, threadfin breams |  | <$2 | na | na | $17-25 | na |
| *Nemipterus zysron* | Slender threadfin bream | M |  |  |  |  |  |
| *Nemipterus* sp. | threadfin bream | M |  |  |  |  |  |
| *Pentapodus caninus* | smalltoothed whiptail | M |  |  |  |  |  |
| *Pentapodus setosus* | butterfly whiptail | M |  |  |  |  |  |
| *Scolopsis monogramma* | monogram monocle bream | S |  |  |  |  |  |
| *Scolopsis temporalis (possibly S. monogramma)* | rainbow monocle bream | M |  |  |  |  |  |
|  |  |  |  |  |  |  |  |
| Lethrinidae | emperors, breams |  | <$1-9 | na | na | na | na |
| *Gymnocranius microdon* | bluespotted large-eye bream | M, S |  |  |  |  |  |
| *Gymnocranius* sp. | eyebrow large-eye bream | M |  |  |  |  |  |
| *Lethrinus atkinsoni* | yellow tail emperor | M, S |  |  |  |  |  |
| *Lethrinus erythracanthus* | orange-spotted emperor | M |  |  |  |  |  |
| *Lethrinus erythropterus* | longfin emperor | S |  |  |  |  |  |
| *Lethrinus harak* | thumbprint emperor | M, S |  |  |  |  |  |
| *Lethrinus lentjan* | pinkear emperor | M, S |  |  |  |  |  |
| *Lethrinus microdon* | smalltooth emperor | M |  |  |  |  |  |
| *Lethrinus obsoletus* | orange-striped emperor | M, S |  |  |  |  |  |
| *Lethrinus olivaceous* | longface emperor | M, S |  |  |  |  |  |
| *Lethrinus ornatus* | ornate emperor | M, S |  |  |  |  |  |
| *Lethrinus rubrioperculatus* | spotcheck emperor | M, S |  |  |  |  |  |
| *Lethrinus semicinctus* | blackblotch emperor | S |  |  |  |  |  |
| *Lethrinus xanthochilus* | yellowlip emperor | M |  |  |  |  |  |
| *Lethrinus* sp. | emperor | M |  |  |  |  |  |
|  |  |  |  |  |  |  |  |
| Siganidae | rabbitfishes |  | $0.15-3 | below | below | below | below |
| *Siganus argenteus* | forktail rabbitfish | M |  |  |  |  |  |
| *Siganus corallinus* | coral rabbitfish | M, S |  |  |  |  |  |
| *Siganus guttatus* | golden rabbitfish | M, S |  |  |  |  |  |
| *Siganus javus* | Java rabbitfish | M |  |  |  |  |  |
| *Siganus puellus* | masked rabbitfish | M, S |  |  |  |  |  |
| *Siganus punctatus* | gold-spotted rabbitfish | M, S |  |  |  |  |  |
| *Siganus vermiculatus* | vermiculate rabbitfish | M |  |  |  |  |  |
| *Siganus virgatus* | virgate rabbitfish | M, S |  |  |  |  |  |
| *Siganus vulpinus* | foxface | M, S | $0.15 | $1.50 | $30-90 | $30-45 | $38-78 |
|  |  |  |  |  |  |  |  |
| Ostraciidae | boxfishes |  | $1 | na | na | $30-70 | $26-45 |
| *Lactoria fornasini* | thornback cowfish | M |  |  |  |  |  |
| *Rhynchostracion nasus* | shortnose boxfish | M |  |  |  |  |  |
| *Rhynchostracion rhinorhynchus* | spotted box | M |  |  |  |  |  |
|  |  |  |  |  |  |  |  |
| Diodontidae | porcupinefishes |  |  | $1.50 | $40-149 | $30-60 | na |
| *Diodon histrix* | porcupinefish | S | free |  |  |  |  |
|  |  |  |  |  |  |  |  |
| Monacanthidae | filefishes |  |  |  |  |  |  |
| *Aluterus scriptus* | scrawled filefish | M, S | $1 | na | $15-80 | $15-190 | $21-28 |
|  |  |  |  |  |  |  |  |
| Balistidae | triggerfishes |  | $1-2 | na | $15-140 | $20-60 | $37-140 |
| *Abalistes stellatus* | starry triggerfish | M, S |  |  |  |  |  |
| *Odonus niger* | redtooth triggerfish | M, S |  |  |  |  |  |
|  |  |  |  |  |  |  |  |
| Sciaenidae | croakers |  | na | na | na | na | na |
| *Otolithes ruber* | tigertooth croaker | S |  |  |  |  |  |
|  |  |  |  |  |  |  |  |
| Acanthuridae | surgeonfishes |  | $1-3 | $1-10 | $49-89 | $30-40 | $30-40 |
| *Acanthurus mata* | yellowmask surgeonfish | M, S |  |  |  |  |  |
| *Acanthurus olivaceus** | orangeband surgeonfish | S |  |  |  |  |  |
| *Ctenochaetus striatus* | lined/striped bristletooth | M |  |  |  |  |  |
| *Naso brachycentron* | humpback unicornfish | M, S |  |  |  |  |  |
| *Naso lituratus** | orangespine surgeonfish | S |  |  |  |  |  |
| *Naso lopezi* | slender unicornfish | M |  |  |  |  |  |
| *Naso thynnoides* | barred unicornfish | M |  |  |  |  |  |
| *Naso tuberosus* | humpnose unicornfish | M |  |  |  |  |  |
| *Naso unicornis* | bluespined unicornfish | M |  |  |  |  |  |
|  |  |  |  |  |  |  |  |
| Pomacanthidae | angelfishes |  | na | $3.75 | $19-399 | $40-200 | $18-168 |
| *Pomacanthus imperator** | emperor angelfish | S |  |  |  |  |  |
| *Pomacanthus sexstriatus** | six-banded angelfish | M |  |  |  |  |  |
|  |  |  |  |  |  |  |  |
| Haemulidae | grunts, sweetlips |  | $2-6 | $1.30-2.25 | $60 | na | $14-46 |
| *Diagramma melanacrum* | blacktip/Indonesian sweetlips | S |  |  |  |  |  |
| *Plectorhinchus chaetodonoides* | many-spotted sweetlips | M |  |  |  |  |  |
| *Plectorhinchus flavomaculatus* | goldspotted sweetlips | M |  |  |  |  |  |
| *Plectorhinchus lessonii* | striped sweetlips | S |  |  |  |  |  |
| *Plectorhinchus lineatus* | oriental sweetlips | M |  |  |  |  |  |
| *Plectorhinchus polytaenia* | ribbon sweetlips | M, S |  |  |  |  |  |
| *Pomadasys argenteus* | silver grunt | M |  |  |  |  |  |
| *Pomadasys kaakan* | javelin grunt | M, S |  |  |  |  |  |
|  |  |  |  |  |  |  |  |
| Lutjanidae | snappers |  | $2-5 | na | below | $25-35 | below |
| *Lutjanus bohar* | red snapper | M, S |  |  |  |  |  |
| *Lutjanus boutton* | button snapper | S |  |  |  |  |  |
| *Lutjanus decussatus* | checkered snapper | M, S |  |  |  |  |  |
| *Lutjanus ehrenbergii* | blackspot/Ehrenberg's snapper | M |  |  |  |  |  |
| *Lutjanus fulviflamma* | longspot snapper | S |  |  |  |  |  |
| *Lutjanus gibbus* | humpback snapper | M, S |  |  |  |  |  |
| *Lutjanus kasmira** | bluestripe snapper | S |  |  |  |  |  |
| *Lutjanus timorensis* | Timor snapper | S |  |  |  |  |  |
| *Macacolor macularis* | midnight snapper | M |  |  |  |  |  |
| *Symphorus nematophorus* | chinamanfish | S | $5 | na | $90 | $90 | $71-124 |
|  |  |  |  |  |  |  |  |
| Caesionidae | fusiliers |  | <<$1 | na | na | na | na |
| *Caesio caerulaurea* | scissortail/blue-and-gold fusilier | M |  |  |  |  |  |
| *Caesio cuning* | yellowtail fusilier | M, S |  |  |  |  |  |
| *Pterocaesio chrysozona* | goldband fusilier | M |  |  |  |  |  |
| *Pterocaesio digramma* | doublelined fusilier | M, S |  |  |  |  |  |
| *Pterocaesio tesselatta* | narrowstripe fusilier | M |  |  |  |  |  |
| *Pterocaesio tile* | bluestreak fusilier | M, S |  |  |  |  |  |
|  |  |  |  |  |  |  |  |
| Kyphyosidae | drummers, chubs |  |  |  |  |  |  |
| *Kyphosus cinerascens* | topsail drummer | M, S |  |  |  |  |  |
| *Kyphosus vaigiensis (K. bigibbus?)* | lowfin/brassy drummer | S |  |  |  |  |  |
|  |  |  |  |  |  |  |  |
| Serranidae | groupers, coral trouts |  | $2-7 | $0.59-2.25 | $27-200 | $30-50 | $30-50 |
| *Anyperodon leucogrammicus* | slender grouper | S |  |  |  |  |  |
| *Cephalopholis argus* | peacock grouper | M |  |  |  |  |  |
| *Cephalopholis miniata* | coral grouper/cod/trout | M, S |  |  |  |  |  |
| *Cephalopholis sonnerati* | tomato grouper | M, S |  |  |  |  |  |
| *Epinephelus aureolatus* | aureolate grouper | S |  |  |  |  |  |
| *Epinephelus corallicola* | coral rock grouper | M, S |  |  |  |  |  |
| *Epinephelus fasciatus* | blacktip grouper | M |  |  |  |  |  |
| *Epinephelus fuscoguttatus* | brown-marbled grouper | M |  |  |  |  |  |
| *Epinephelus macrospilos* | snubnose grouper | M, S |  |  |  |  |  |
| *Epinephelus ongus* | whitestreaked grouper | S |  |  |  |  |  |
| *Epinephelus sexmaculata* | saddled grouper | M |  |  |  |  |  |
| *Epinephelus socialis* | surge grouper | S |  |  |  |  |  |
| *Plectropomus leopardus* | leopard coral grouper | S, M |  |  |  |  |  |
| *Plectropomus maculatus* | spotted coral grouper | M |  |  |  |  |  |
| *Plectropomus oligacanthus* | highfin coral grouper | M |  |  |  |  |  |
| *Variola albimarginata* | lyretail grouper | M, S |  |  |  |  |  |
| *Variola louti* | yellowmargin lyretail | M |  |  |  |  |  |
|  |  |  |  |  |  |  |  |
| Platycephalidae | flatheads |  | <$1 | na | na | na | na |
| *Cymbacephalus* sp. | crocodile fish ('ikan buaya') | M |  |  |  |  |  |
| *Inegocia japonica* | Japanese flathead | S |  |  |  |  |  |
|  |  |  |  |  |  |  |  |
|  |  |  |  |  |  |  |  |

| **Ornamental Fishes** |  |  |  |  |
| --- | --- | --- | --- | --- |
| Family and Species | Common Name | # Individuals | Value | Size |
| Apogonidae |  |  |  |  |
| *Apogon* sp. | ‘pterapogon kaudlrnii'/Banggai cardinalfish | 62 | $1.95 | M |
| *Zoramia leptacantha* | ‘longspine'/threadfin cardinalfish | 24 | $0.50 | M |
|  |  |  |  |  |
| Gobiidae |  |  |  |  |
| *Amblygobius phalaena* | banded goby | 2 | $0.70 | M |
| *Cryptocentrus cinctus* | ‘yellow lizardfish'/yellow shrimpgoby | 4 | $1.50 | M |
| *Cryptocentrus leptocephalus* | ‘pink and blue spotted goby'/pink-speckled shrimpgoby | 13 | $0.95 | M |
| *Signigobius biocellatus* | ‘two spot'/twinspot goby | 6 | $1.50 | M |
| *Tomiyamichthys oni* | ‘mottled'/monster shrimpgoby | 2 | $0.99 | M |
| *Valenciennea helsdingenii* | ‘two stripe sleeper'/two-stripe goby | 6 | $0.99 | M |
| *Valenciennea strigata* | ‘golden _ads'/blueband goby | 3 | $0.99 | M |
| *Stonogobiops nematodes* | ‘black stripe'/blackrayed shimpgoby | 6 | $1.95 | M |
|  |  |  |  |  |
| [Callionymidae](http://www.fishbase.org/summary/FamilySummary.php?ID=435) |  |  |  |  |
| *Pterosynchiropus splendidus* | green mandarinfish | 21 | $1.75 | M |
|  |  |  |  |  |
| Syngnathidae |  |  |  |  |
| *Corythoichthys intestinalis* | ‘Australian banded goby'/messmate/scribbled pipefish | 11 | $0.95 | M |
| *‘Doryrhamphus dactyliophorus’*  *(Dunckerocampus dactyliophorus)* | ‘banded goby'/ringed pipefish | 12 | $0.90 | M |
| *Syngnathoides biaculeatus* | ‘alligator goby'/double-ended pipefish/alligator pipefish | 3 | $0.60 | M |
|  |  |  |  |  |
| Sphyraenidae |  |  |  |  |
| *Sphyraena barracuda* | great barracuda | 3 | $2.50 | M |
|  |  |  |  |  |
| Pomacentridae |  |  |  |  |
| *Amphiprion clarkii* | Clark's anemonefish | 16 | $0.69 | M |
| *Amphiprion melanopus* | ‘tomato clownfish'/red and black anemonefish | 14 | $0.69 | M |
| *Amphiprion ocellaris* | ‘clown anemonefish'/false clownfish | 79 | $0.75 | M |
| *Amphiprion sandaracinos* | orange skunk clownfish | 2 | $0.69 | M |
| *Amphiprion sebae* | sebae anemonefish | 10 | $0.69 | M |
| *Chromis viridis* | green chromis | 200 | $0.25 | M |
| *Chrysiptera cyanea* | ‘blue damsel'/blue devil | 220 | $0.25 | M |
| *Premnas biaculeatus* | ‘maroon clown'/spinecheek anemonefish | 11 | $0.75 | M |
| *Pomacentrus caeruleus* | ‘kupang damsel'/bluedevil/caerulean damselfish | 30 | $0.90 | M |
| *Pomacentrus* sp. | ‘rid damsel' | 1 | $0.59 | M |
|  |  |  |  |  |
| Blenniidae |  |  |  |  |
| *Ecsenius bicolor* | ‘two colored goby'/bicolor blenny | 3 | $1.19 | M |
| *Salarias fasciatus* | ‘banded blenny'/jeweled blenny | 22 | $0.69 | M |
|  |  |  |  |  |
| Labridae |  |  |  |  |
| *Cirrhilabrus cyanopleura* | ‘blue fairy'/bluesided wrasse | 5 | $0.60 | M |
| *Cirrhilabrus lubbocki* | ‘turbock's fairy fish'/Lubbock's wrasse | 6 | $3.50 | M |
| *Halichoeres chrysus* | ‘yellow coris'/canary wrasse | 27 | $0.85 | M |
| *Halichoeres hortulanus* | ‘basket fish'/checkerboard wrasse | 9 | $0.60 | M |
| *Paracheilinus* sp. | ‘vei vei fish'/flasherwrasse | 31 | $0.50 | M |
| *Pseudocheilinus hexataenia* | ‘six line fish'/sixline wrasse | 1 | $0.99 | M |
| *Thalassoma lunare* | ‘lytretail wrasse'/moon wrasse | 3 | $0.70 | M |
|  |  |  |  |  |
| Scaridae |  |  |  |  |
| *Cetoscarus bicolor* | bicolor parrotfish | 6 | $2.25 | M |
|  |  |  |  |  |
| [Ephippidae](http://www.fishbase.org/summary/FamilySummary.php?ID=340) |  |  |  |  |
| *Platax pinnatus* | longfinned batfish | 3 | $6.90 | M |
|  |  |  |  |  |
| Siganidae |  |  |  |  |
| *Siganus vulpinus* | foxface rabbitfish | 3 | $1.50 | M |
|  |  |  | |  |
| Diodontidae |  |  |  |  |
| *Diodon holocanthus* | ‘porcupine puffer'/longspined porcupinefish/ballonfish | 1 | $1.50 | M |
|  |  |  |  |  |
| [Tetraodontidae](http://www.fishbase.org/summary/FamilySummary.php?ID=448) |  |  | |  |
| *Canthigaster* sp. | ‘assorted valentini'/puffers | 12 | $0.85 | M |
|  |  |  |  |  |
| Acanthuridae |  |  |  |  |
| *Acanthurus chronixis* | mimic tang | 6 | $0.90 | M |
| *Acanthurus leucosternon* | powderblue surgeonfish | 32 | $8.99 | M/L |
| *Naso lituratus* | orangespine unicornfish | 6 | $9.90 | M |
| *Paracanthurus hepatus* | ‘blue tang'/palette surgeonfish | 7 | $7.99 | M |
| *Zebrasoma velifer* | sailfin tang | 3 | $2.99 | L/M |
|  |  |  |  |  |
| Chaetodontidae |  |  |  |  |
| *Chaetodon baronessa* | ‘barones's'/eastern triangular butterflyfish | 3 | $0.99 | M |
| *Chaetodon kleinii* | ‘Klein's'/sunburst butterflyfish | 1 | $0.99 | M |
| *Chelmon rostratus* | ‘copperband' butterflyfish/beaked coralfish | 26 | $1.35 | M |
|  |  |  |  |  |
| Pomacanthidae |  |  |  |  |
| *Apolemichthys trimaculatus* | ‘hagfin'/threespot angelfish | 1 | $4.95 | L/M |
| *Centropyge bicolor* | bicolor angelfish | 10 | $1.59 | M |
| *Centropyge flavicauda* | ‘blue pygmy angel'/whitetail angelfish | 13 | $0.99 | M |
| *Centropyge tibicen* | ‘meias'/keyhole angelfish | 10 | $0.99 | M |
| *Centropyge vroliki* | 'half black'/pearlscale angelfish | 9 | $0.99 | M |
| *Euxiphipops xanthometopon* | ‘blue mask'/yellowmask angelfish | 1 | $28.99 | M |
| *Pomacanthus imperator* | emperor angelfish | 2 | $12.95 | M |
| *Pomacanthus sexstriatus* | sixbar angelfish | 4 | $3.75 | M |
|  |  |  |  |  |
| Haemulidae |  |  |  |  |
| *Plectorhinchus chaetodonoides* | ‘spotted grunt'/many-spotted sweetlips | 13 | $2.25 | L/M |
| *Plectorhinchus diagrammus* | striped sweetlips | 1 | $1.30 | M |
| *Plectorhinchus picus* | painted sweetlips | 1 | $1.30 | M |
|  |  |  |  |  |
| Cirrhitidae |  |  |  |  |
| *Cirrhitichthys falco* | ‘falco'/dwarf hawkfish | 11 | $0.70 | M |
|  |  |  |  |  |
| Serranidae |  |  |  |  |
| *Cromileptes altivelis* | ‘panther grouper'/barramundi cod/humpback grouper | 6 | $2.25 | M |
| *Pseudanthias dispar* | ‘madder seaperch'/redfin anthias | 18 | $0.59 | M |
| *‘Pseudanthias hutchi' (P. huchtii)* | ‘sea goldie'/red-cheeked anthias | 14 | $0.59 | M |
| *Pseudanthias pleurotaenia* | ‘yellow anthias'/squarespot anthias | 10 | $2.35 | M |
| *Pseudanthias squamipinnis* | ‘lyre tail anthias'/scalefin anthias/sea goldie | 16 | $0.99 | M |
| *Odontanthias borbonius* | ‘bourbon fairy basslet'/checked swallowtail | 6 | $60.00 |  |
|  |  |  |  |  |
| Scorpaenidae |  |  |  |  |
| *Dendrochirus zebra* | ‘dwarf lionfish'/zebra turkeyfish | 5 | $0.70 | M |
| *Pterois volitans* | ‘red volitans'/lionfish | 3 | $2.99 | M |
